# Osteoclast-like giant cell tumor of the parotid gland; a case report with literature review

**DOI:** 10.1016/j.amsu.2022.103509

**Published:** 2022-03-28

**Authors:** Abdulwahid M. Salih, Berwn A. Abdulla, Ari M. Abdullah, Fahmi H. Kakamad, Zana H. Hassan, Razhan K. Ali, Suhaib H. Kakamad

**Affiliations:** a College of Medicine, University of Sulaimani, Sulaimani, Iraq; b Smart Health Tower, Madam Mitterrand Street, Sulaimani, Kurdistan, Iraq; c Kscien Organization, Hamdi Str, Azadi Mall, Sulaimani, Kurdistan, Iraq; d Sulaimani Teaching Hospital, Sulaimani, Kurdistan, Iraq; e College of Dentistry, University of Sulaimani, Sulaimani, Iraq; f Shar Hospital, College of Medicine, Sulaimani, Iraq

**Keywords:** Giant cell tumor, Parotid gland, Osteoclast-like giant cell tumor, Salivary gland

## Abstract

**Introduction:**

The osteoclast-like giant cell is a benign tumor that presents as either an isolated benign tumor or one with a carcinomatous component. This study aims to report a rare case of osteoclast-like giant cell tumor (GCT) of the parotid gland.

**Case report:**

A 67-year-old female presented with a painless left pre-auricular swelling of 2-month duration which increased in size gradually over that period. On examination, there was a firm, mobile mass with well-defined borders in the left parotid gland. Fine needle aspiration cytology showed a giant cell-rich lesion that was highly cellular and contained a large number of osteoclast-like multinucleated giant cells, with clusters of spindle and epithelioid cells. Total parotidectomy was performed. After the operation, the patient was sent for radiotherapy.

**Discussion:**

The histogenesis and exact nature of this tumor are unknown although numerous ideas have been put forward. The most common clinical manifestation is a painless slow-growing tumor in the parotid area. Primary osteoclast-like GCT of the salivary gland might show concomitant benign or malignant neoplasms. There is also a “pure form” of the tumor that has no accompanying neoplasm.

**Conclusion:**

GCT of the parotid gland is a rare tumor. The histogenesis and nature of parotid gland GCT are not completely understood. The treatment of choice is total excision followed by radiotherapy.

## Highlights

• Giant cell tumors of the parotid gland are rare tumors.
• The most common clinical manifestation is a painless slow-growing mass.
• It can be in a pure form or a mix of benign and malignant components.

## 1 Introduction

Giant cell tumor (GCT) of bone is a benign osteolytic bone tumor that is filled with osteoclast-like multinucleated giant cells (MGCs) and contributes to about 5% of all primary bone tumors in adults. It leads to extensive bone destruction [1,2]. The abnormal process of osteoclastogenesis in the GCT of bone has an unexplained underlying mechanism. The origin of a GCT is typically thought to be the bone, but it has also been found in the pancreas, thyroid, lung, liver, breast, ovary, and kidney. It has also been observed among other soft tissue and parenchymal organs, such as the parotid and salivary glands [3,4]. Osteoclast-like GCT of the parotid is a benign soft tissue tumor that has the potential to become malignant. It is uncommon and only a few cases have been recorded in literature [4]. They have been thought to be either a part of a stromal process reacting to neoplasia or a component of a primary neoplasm; however, their exact etiology is unclear [5]. There have been two types of presentations: either an isolated GCT or a tumor with a carcinomatous component [6,7]. This study aims to present a rare case of extraosseous GCT of the major salivary gland. The report has been written in line with SCARE 2020 guidelines [8].

## 2 Case report

**Patient Information:** A 67-year-old female presented with a painless left pre-auricular swelling of 2-month duration which increased in size over that period. The patient was diabetic and hypertensive, and hence, was taking anti-diabetic and anti-hypertensive medications. Her past medical history aside from that was not significant.

**Clinical Findings:** On examination, there was a painless, firm, and mobile mass with well-defined borders in the left parotid gland. No skin changes or ulceration over the mass were observed.

**Diagnostic Assessment:** Hematological examinations revealed HbA1c of 7.7%, a blood sugar level of 227mg/dl, and an inflammatory marker (CRP) of 3.18 mg/l. On ultrasound examination, there was a well-defined hypoechoic solid nodule of about 33 × 27*11 mm in the left preauricular region. The nodule was in contact with the ramus of the mandible and showed malignant characteristics - along with a 7mm short axis lymph node seen in the left infra-auricular region without pathological sonographic characteristics. No obvious pathological lymph node was seen elsewhere in the neck.

Fine needle aspiration cytology (FNAC) was performed which showed a giant cell-rich lesion that was highly cellular and contained a large number of osteoclast-like multinucleated giant cells with clusters of spindle and epithelioid cells (stroma) and a few lobulated benign-looking acini of parotid glands.

The patient was sent for contrast magnetic resonance (MRI) imaging of the neck which revealed a single well-defined lesion in the superficial lobe in the left parotid gland measuring about 33 × 20 mm and showing a heterogeneous signal on T1 and T2, suggesting mostly pleomorphic adenoma. The right parotid gland was normal with bilateral benign-looking cervical lymph nodes of 6mm in the short axis - with normal submandibular and thyroid glands. The pharynx and larynx were normal.

**Fig. 1:**
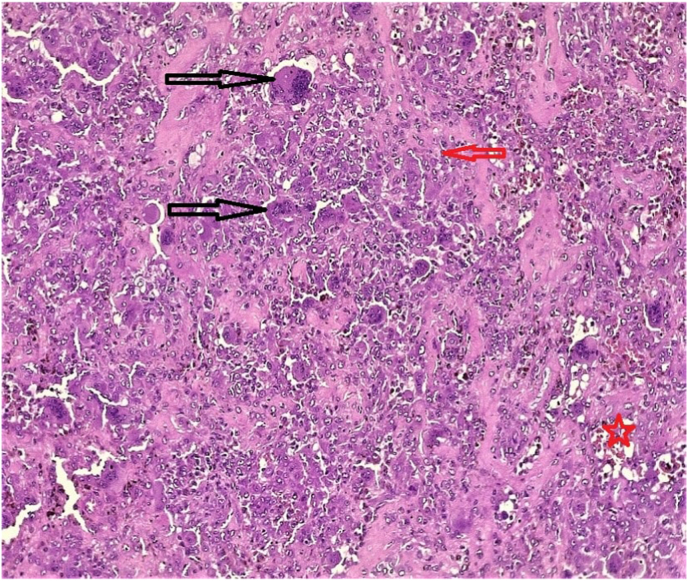
Microscopic photo of the specimen revealing a well demarcated mass composing of evenly distributed osteoclast-like multinucleated giant cells (black arrows) intermixed with other population of mono-nuclear spindle cells (red arrow) having abundant eosinophilic cytoplasm, low mitotic count (1–3 mitoses/10 HPF) with brown pigment deposition (red star). (For interpretation of the references to colour in this figure legend, the reader is referred to the Web version of this article.)

**Therapeutic Intervention:** Superficial-deep parotidectomy was performed on the patient. The result of histopathological examination revealed a giant cell tumor of 3.4 cm without any lymph node involvement (Fig. 1). The tumor resection margin was 1.5 cm. After the operation, the patient was sent for radiotherapy.

**Follow up:** the patient was well and healthy six months after the operation.

## 3 Discussion

A GCT is a lesion that develops from undifferentiated mesenchymal cells in the bone marrow and has a marginal or ambiguous biological activity. This, however, is not recognized as a salivary gland tumor by the World Health Organization (WHO). The histogenesis of this tumor is unknown although numerous hypotheses have been put forward [9]. We could collect only 16 cases from the literature, indicating that GCT of the parotid gland is very uncommon. Eusebi was the first to describe the GCT of the parotid gland [6].

The most common clinical manifestation is a painless slow-growing tumor in the parotid area [10]. The patients in the cases gathered by Deligiannidis et al. ranged in age from 24 to 92 years old, and the tumors were found to be more common in men than in women; the mean age of occurrence was 56 years [11].

In previous incidents, a link between primary osteoclast-like GCT of the salivary gland and concomitant benign and malignant neoplasms was reported [9,12].

There is also a “pure form” of the tumor that has no accompanying malignancy [13]. Because malignancy was identified in the majority of the patients, the frequency with which parotid gland GCT coexists with malignant neoplasms is of particular interest [9].

The parotid gland, mandible, or soft tissues around the parotid gland can all cause GCT in the parotid area. The so-called GCT of the parotid gland appears to share some features with other GCTs, hinting that they have a separate etiology.

The first feature is the tumor's link to neighboring anatomical components, as shown intraoperatively because extra-parotid tumors appear to be a continuation of their tissues of origin, whereas parotid tumors are distinct from the parotid [14]. Furthermore, unlike GCT of the bone, there is no reactive bone production in the periphery of parotid gland tumors [14]. Finally, they exhibit more aggressive biological behavior, which has been linked to a higher prevalence of concomitant malignancy [9].

Based on the expression of epithelial and histiocytic markers from mononuclear cells, as well as their higher coincidence with malignant findings, some investigators claim that GCT of the parotid gland has an epithelial origin and looks more like carcinomas [9]. Despite efforts to shed light on the matter, the specific cause of this tumor remains unknown.

The recommended treatment, despite the vague nature of the tumor, is total excision of the lesion with healthy tissue margins to reduce the likelihood of recurrence, particularly those with associated malignancy. For patients with poor resection margins or those who cannot undergo surgery owing to underlying problems, radiotherapy can be considered to play a role in the treatment of these lesions [10].

In conclusion, GCT of the parotid gland is a rare tumor. The histogenesis and nature of parotid gland GCT are not completely understood. It differs from GCT of bone in that it is not a continuation of neighboring tissues and there is no reactive bone production. The treatment of choice is total excision followed by radiotherapy.

## Sources of funding

None is found.

## Consent

Written informed consent was obtained from the patient for publication of this case report and accompanying images. A copy of the written consent is available for review by the Editor-in-Chief of this journal on request.

## Research registration

Not applicable.

## Ethical Approval

N/A.

## Author statement

Abdulwahid M. Salh: Surgeon performing the operation, final approval of the manuscript.

Zana H. Hassan: literature review, writing the manuscript, final approval of the manuscript.

Razhan K. Ali, Suhaib H. Kakamad, Berwn A. Abdulla, Fahmi H. Kakamad: literature review, review and final approval of the manuscript.

Ari M. Abdullah: The histopathologist who made the diagnosis, final approval of the manuscript.

## Guarantor

Fahmi Hussein Kakamad is Guarantor of this submission.

## Registration of Research Studies

N/A.

## Declaration of competing interest

None to be declared.
